# The Effect of Mindfulness Interventions for Parents on Parenting Stress and Youth Psychological Outcomes: A Systematic Review and Meta-Analysis

**DOI:** 10.3389/fpsyg.2019.01336

**Published:** 2019-06-06

**Authors:** Virginia Burgdorf, Marianna Szabó, Maree J. Abbott

**Affiliations:** School of Psychology, The University of Sydney, Sydney, NSW, Australia

**Keywords:** mindfulness, mindful parenting, parenting intervention, parenting stress, child externalizing, child internalizing, meta-analysis, systematic review

## Abstract

**Background:** The psychological well-being of parents and children is compromised in families characterized by greater parenting stress. As parental mindfulness is associated with lower parenting stress, a growing number of studies have investigated whether mindfulness interventions can improve outcomes for families. This systematic review and meta-analysis evaluates the effectiveness of mindfulness interventions for parents, in reducing parenting stress and improving youth psychological outcomes.

**Methods:** A literature search for peer-reviewed articles and dissertations was conducted in accordance with PRISMA guidelines in the PsycInfo, Medline, PubMed, CINAHL, Web of Science, Cochrane Central Register of Controlled Trials, and ProQuest Dissertations & Theses databases. Studies were included if they reported on a mindfulness-based intervention delivered in person to parents with the primary aim of reducing parenting stress or improving youth psychological outcomes.

**Results:** Twenty-five independent studies were included in the review. Eighteen studies used a single group design and six were randomized controlled trials. Within-groups, meta-analysis indicated a small, post-intervention reduction in parenting stress (*g* = 0.34), growing to a moderate reduction at 2 month follow-up (*g* = 0.53). Overall, there was a small improvement in youth outcomes (*g* = 0.27). Neither youth age or clinical status, nor time in mindfulness training, moderated parenting stress or overall youth outcome effects. Youth outcomes were not moderated by intervention group attendees. Change in parenting stress predicted change in youth externalizing and cognitive effects, but not internalizing effects. In controlled studies, parenting stress reduced more in mindfulness groups than control groups (*g* = 0.44). Overall, risk of bias was assessed as serious.

**Conclusions:** Mindfulness interventions for parents may reduce parenting stress and improve youth psychological functioning. While improvements in youth externalizing and cognitive outcomes may be explained by reductions in parenting stress, it appears that other parenting factors may contribute to improvements in youth internalizing outcomes. Methodological weaknesses in the reviewed literature prevent firm conclusions from being drawn regarding effectiveness. Future research should address these methodological issues before mindfulness interventions for parents are recommended as an effective treatment option for parents or their children.

## Introduction

Parenting stress is associated with negative outcomes for parents and their children (Davis and Carter, 2008; Deater-Deckard et al., 2016). Recently, several studies have linked lower parenting stress with higher parental mindfulness (e.g., Parent et al., 2016; Campbell et al., 2017). Accordingly, a growing number of studies have delivered mindfulness-based interventions to parents, with the aim of reducing parenting stress and improving psychological outcomes for youth (e.g., Zhang et al., 2017; Jones et al., 2018). However, no quantitative synthesis of the literature on the effectiveness of such interventions is currently available. This review and meta-analysis was conducted to evaluate the effectiveness of mindfulness interventions for parents, in reducing parenting stress and improving youth psychological outcomes.

Parents who experience higher parenting stress report poorer psychological well-being (Lavee et al., 1996), more negative affect and less positive affect (Deater-Deckard et al., 2016), and lower marital quality (Robinson and Neece, 2015). In families characterized by greater parenting stress, children have more internalizing and externalizing problems (Huth-Bocks and Hughes, 2007; Davis and Carter, 2008; Robinson and Neece, 2015), poorer cognitive skills such as executive function (de Cock et al., 2017) and more social and interpersonal difficulties (Anthony et al., 2005). Greater parenting stress is also associated with negative parenting behaviors, including harsh discipline (Venta et al., 2016) and hostility (McMahon and Meins, 2012), which have been shown to contribute to poorer child and adolescent psychological outcomes (Rominov et al., 2016; Pinquart, 2017). Managing parenting stress is therefore important for the well-being of parents and their children. It has been suggested that incorporating mindfulness into the parent-child relationship may be one way of achieving this goal (Kabat-Zinn and Kabat-Zinn, 1997; Dumas, 2005; Duncan et al., 2009; Bögels et al., 2010).

In the context of contemporary Western psychology, mindfulness is typically described as a psychological process of bringing non-judgmental awareness to experiences occurring in the present moment (Kabat-Zinn, 2015). Individuals differ in their disposition for mindfulness but can develop their skills through regular practice (Kabat-Zinn, 2003, 2015; Baer et al., 2006). The application of mindfulness to parenting was first described by Kabat-Zinn and Kabat-Zinn (1997). These authors defined mindful parenting as paying non-judgmental, non-reactive attention to each moment and interaction with the child, such that the parent is aware of their child's needs in any moment. Building on this account, Duncan et al. (2009) developed a model of mindful parenting comprising five dimensions: listening to the child with full attention, non-judgmental acceptance of self and child, emotional awareness of self and child, self-regulation in parenting, and compassion for self and child. Mindful parents reduce their use of automatic but unhelpful ways of evaluating or interacting with their child, thus making way for more positive parent-child relationships (Dumas, 2005; Duncan et al., 2009). For example, mindfulness can assist parents to break a habitual pattern of automatically reacting with anger to a child's tantrum, which is likely to elicit further negative affect from the child (Dumas, 2005).

In light of these ideas, mindfulness-based interventions such as the 8-week Mindfulness-based Stress Reduction program (MBSR; Kabat-Zinn et al., 1992), have been offered to parents who experience high levels of stress, anxiety, or depression (Bazzano et al., 2015). Other researchers have adapted the MBSR program specifically to the parenting context (Bögels et al., 2014; Eames et al., 2015). These mindful parenting programs are based upon the same principles of mindfulness as MBSR and follow a similar session structure. MBSR for parents and mindful parenting programs both aim to improve outcomes for families, particularly reducing parenting stress (for example, Neece, 2014; Chaplin et al., 2018). However, mindful parenting programs focus specifically on the stressors faced by parents and the patterns of interaction they have with their children. For example, the well known “observing a raisin” exercise is used in MBSR to illustrate the concept of stepping out of automatic pilot. In one mindful parenting course (Bögels and Restifo, 2014), this exercise is followed by a homework practice in which parents mindfully observe their child, using the skills they learnt while observing a raisin.

In the past decade, a number of studies have explored the effects of both MBSR and mindful parenting interventions on parenting stress. Following MBSR programs, reductions in parenting stress were reported by parents of pre-school aged children with Autism Spectrum Disorder (ASD) and other developmental delays (Chan and Neece, 2018). In a similar clinical sample, the reductions in parenting stress were larger for the MBSR group than a waitlist control group (Neece, 2014). Mindful parenting interventions have been offered in community, as well as in clinical settings. In two small studies of community-recruited parents, no reduction in parenting stress was found following mindful parenting training (Maloney and Altmaier, 2007; Eames et al., 2015), whilst in a larger community study, a reduction was reported (Potharst et al., 2018). The difference in sample sizes may account for the contrasting findings in these studies. In the clinical context, parents of children and adolescents with a range of externalizing and internalizing disorders (Bögels et al., 2014; Ridderinkhof et al., 2017) reported both immediate and maintained reductions in parenting stress following mindful parenting interventions. In contrast, parents of children with Attention Deficit and Hyperactivity Disorder (ADHD) reported a moderate reduction in parenting stress only at 2 month follow-up (van der Oord et al., 2012). The majority of mindful parenting intervention studies have used a single group design. However, a small number of controlled studies have found mindful parenting groups report greater reductions in parenting stress than control groups, in community and clinical settings (Ferraioli and Harris, 2013; Lo et al., 2017a; Corthorn, 2018). In sum, although results are mixed, MBSR and mindful parenting interventions appear to be associated with reduced levels of parenting stress, both in community and clinical contexts.

Studies of MBSR and mindful parenting have also investigated outcomes for the children of parents who attended the interventions. Most studies investigated internalizing and externalizing symptoms, which are the most common psychological problems in youth (Bayer et al., 2008). A number of studies also examined cognitive and social domains of functioning, both of which are related to important longer term problems, such as poorer academic achievement (Malecki and Elliott, 2002; Daley and Birchwood, 2010). Following their parents' attendance at MBSR, pre-school aged children with ASD and other developmental delays showed significant improvements in cognitive, externalizing, and social outcomes (Neece, 2014; Lewallen and Neece, 2015). Following mindful parenting training, small to moderate reductions in youth internalizing problems have been reported by youth with a range of mental health problems and their parents (Bögels et al., 2014; Haydicky et al., 2015; Racey et al., 2017). In contrast, in a study involving 10 adolescents with ADHD, no significant improvements in adolescent internalizing problems were reported (van de Weijer-Bergsma et al., 2012). Similarly, externalizing problems have been reported to reduce after mindful parenting interventions by parents (Bögels et al., 2014; Meppelink et al., 2016) and youth (Bögels et al., 2008; Ridderinkhof et al., 2017) in some studies, but not in others (De Bruin et al., 2015; Jones et al., 2018). In relation to cognitive outcomes, parents have reported fewer attention problems (Ridderinkhof et al., 2017), but no reductions in metacognitive (Zhang et al., 2017) or learning problems (Haydicky et al., 2015). Finally, after mindful parenting interventions, youth social outcomes improved in some studies (Bögels et al., 2008; Haydicky et al., 2015) but not others (De Bruin et al., 2015; Jones et al., 2018). The results of the literature relating to youth outcomes are therefore mixed.

Considering the number of studies and the mixed results they report, a quantitative evaluation of the available data is needed. However, there are no published meta-analyses in this field of research. Further, although two narrative reviews have been conducted, neither of these focuses exclusively on mindfulness interventions delivered to parents. Harnett and Dawe (2012) reviewed 24 interventions incorporating mindfulness, for school students and their careers. Only two of those interventions were delivered to parents. Moreover, those two interventions were not primarily mindfulness interventions. Instead, they incorporated an element of mindfulness into existing behavioral skills programs. Townshend et al. (2016) reviewed seven randomized controlled trials (RCTs) of various interventions delivered to parents. Again, only two of the reviewed trials delivered interventions that were primarily mindfulness-based, while the others incorporated aspects of mindfulness in behavioral or emotion-coaching programs. A review focused upon mindfulness interventions for parents is therefore warranted. Accordingly, the aim of this review was to systematically and quantitatively evaluate the effectiveness of mindfulness interventions for parents. To reflect the range of outcomes covered in the existing literature, the outcomes of interest in this review were parenting stress, and youth functioning across internalizing, externalizing, cognitive, and social domains. Due to the noted similarities between mindful parenting interventions and other mindfulness-based interventions such as MBSR for parents, we amalgamated these studies into a single group and will refer to them together as “mindfulness interventions for parents.”

## Methods

The Preferred Reporting Items for Systematic Reviews and Meta-Analyses (PRISMA) statement and checklist (Moher et al., 2009) were used to guide the conduct and reporting of this review.

### Eligibility Criteria

Studies were eligible for inclusion in the review if they reported on a mindfulness-based intervention delivered in person to parents, with a primary aim of reducing parenting stress or improving youth psychological outcomes. Studies that met this criterion that also delivered a parallel mindfulness intervention to a child of the participant parents were included. Studies were excluded if they reported on an intervention that was not a mindfulness-based intervention or if the intervention incorporated other forms of therapy or training such as behavioral parent training, acceptance and commitment therapy or cognitive therapy. Studies were also excluded if they used an individual case series or qualitative design.

### Search Strategy and Information Sources

A comprehensive literature search was conducted between 9 August and 11 October 2018, in the PsycInfo, Medline, PubMed, CINAHL, Web of Science, Cochrane Central Register of Controlled Trials and ProQuest Dissertations & Theses databases, for peer-reviewed articles and published dissertations indexed up to and including 30 September, 2018. In PsycInfo, we searched the database subject headings Mindfulness and Meditation, and the keywords mindful^*^ and meditation, in combination with the subject headings Parenting, Parents, Parenting Style, Parenting Skills, Parental Attitudes, Parent Training, Childrearing Attitudes, Childrearing Practices, Family Intervention and Family Therapy and the key words parent^*^, child?rearing, family intervention^*^, and family therap^*^. For the search, no limitations were placed on the language in which the study was reported. The reference lists of included articles were also searched for relevant studies but no additional studies were identified in this way.

The database search was conducted by the first author. After removal of duplicates, a title and abstract screening of all articles was conducted by the first author to assess the studies against the eligibility criteria. One-third of the articles were also screened independently by a Masters-level graduate student in clinical psychology. A full-text review of the short-listed articles was then conducted independently by both the first author and the same graduate student, with 92% agreement between the two reviewers on the selection of studies for inclusion in the review.

### Data Extraction

All data was extracted by the first author. The data extracted from each study included participant characteristics, youth age and gender, parent and youth psychopathology, study design, and details of the intervention. These study details are presented in Table 1.

**Table 1:** Details of included studies.

**Study**	**Sample size and parents' gender**	**Youth age (range) in years and gender**	**Parent clinical status^**∧**^**	**Youth clinical status and primary diagnosis**	**Study design and conditions**	**Intervention characteristics**		
						**Intervention program**	**Intervention group/s**	**Sessions**
Bazzano et al. (2015)	*N* = 66 parents/caregivers (77% mothers/female)	NR	Non-clinical	Clinical: ASD (59%), ID (21%), cerebral palsy (5%), Down syndrome (3%), other diagnoses (11%)	Uncontrolled trial: 1. MP	MBSR adapted for parents of children with disabilities	Parent/caregiver group	8 weeks × 2 h + 4 h silent retreat; total 20 h
Bögels et al. (2008)	*N* = 14 parents (57% mothers) and 14 adolescents	*M* = 14.4 (11–17) 57% boys	Clinical: DD (21%), PTSD (21%), ADHD (14%), PDD (14%), Asperger's (7%)	Clinical: ODD (43%), PDD (21%), ADHD (14%), CD (14%) ASD (7%)	WLC trial: 1. MP	MBCT adapted for parents	Parent group and separate adolescent mindfulness group	8 weeks × 1.5 h; total 12 h (for both parent and adolescent groups)
Bögels et al. (2014)	*N* = 86 parents (89% mothers)	*M* = 10.7 (2–21) 60% boys	Clinical: Parent-child relational problem (58%), DD (16%), adjustment disorder (8%), BD (2%), ADHD (1%), BPD (1%)	Clinical: ADHD (47%), ASD (21%), AD (7%), DD (5%), ODD (4%), LD (4%), CD (1%), schizophrenia (1%)	WLC trial: 1. MP	MP (Bögels and Restifo, 2013)	Parent group	8 weeks × 3 h; total 24 h
Chan and Neece (2018)#	*N* = 80 parents (96% mothers)	*M* = 4.18 (2.5–5) 71% boys	Non-clinical	Clinical: ASD (64%), other developmental delay (36%)	RCT: 1. MBSR 2. Wait list control	MBSR: MBSR program Control: Nil (offered MBSR program after completion of waitlist period)	MBSR: Parent group Control: Nil	MBSR: 8 weeks × 2 h + 6 h retreat; total 22 h Control: Nil
Chaplin et al. (2018)	*N* = 100 mothers	*M* = 14.04 (12–17) 48% boys	Non-clinical: self-reported parenting stress	Non-clinical: inclusion criteria did not require diagnosis or referral, but 53% of families receiving psychotherapy	RCT: 1. MP 2. Parent education control	MP: Parenting Mindfully (based on MBSR and Duncan et al., 2009)Control: presentation, handouts on adolescent development and parenting, question time	MP: Parent group Control: Parent group	MP: 8 weeks × 2 h; total 16 h Control: 3 meetings × 30 min each
Corthorn (2018)	*N* = 43 mothers	*M* = 2.9 (intervention group) and *M* = 3.0 (control group).Overall range = 2–5Gender NR	Non-clinical	Non-clinical	Controlled trial: 1. MP 2. No treatment control	MP: MBSR adapted for parents Control: Nil	MP: Parent group Control: Nil	MP: 8 weeks × 2 h; total 16 h Control: Nil
De Bruin et al. (2015)	*N* = 29 parents (62% mothers) and 23 adolescents	*M* = 15.8 (11–23) 74% boys	Non-clinical	Clinical: ASD (52%), PDD (48%)	Uncontrolled trial: 1. MP	MP (Bögels and Restifo, 2013)	Parent group and separate adolescent mindfulness group	9 weeks × 1.5 h; total 13 h (for both parent and adolescent groups)
Eames et al. (2015)	*N* = 23 mothers	*M* = 3.14 (1–6) 55% boys	Non-clinical: low socio-economic community	Non-clinical	Uncontrolled trial: 1. MP	Mindfulness-based well-being for parents (adapted from MBSR)	Parent group	8 weeks × 2 h; total 16 h
Ferraioli and Harris (2013)	*N* = 15 parents (66% mothers)	NR (all under 18)	Non-clinical	Clinical: ASD (66%), PDD (34%)	RCT: 1. MP 2. Skills-based parent training Participants matched on parenting stress scores.	MP: Mindfulness-based parent training (adapted from mindfulness module, Linehan, 1993)Control: behavioral parent training for parents of children with ASD	MP: Parent group Control: Parent group	MP: 8 weeks × 2 h; total 16 h Control: 8 weeks × 2 h; total 16 h
Haydicky et al. (2015)	*N* = 17 parents (94% mothers) and 18 adolescents	*M* = 15.5 (13–18) 72% boys	Non-clinical	Clinical: ADHD	WLC trial: 1. MP	MP (adapted from Bögels et al., 2008)	Parent group and separate adolescent mindfulness group	8 weeks × 1.5 h; total 12 h (for both parent and adolescent groups)
Jones et al. (2018)	*N* = 21 parents (86% mothers)	*M* = 10.53 (4–16)Note: mean VABS functioning ability = 4.9562% boys	Non-clinical	Clinical: ASD (76%), ID (10%), cerebral palsy (10%), Down's syndrome (5%)	Uncontrolled trial: 1. MP	Mindfulness-based wellbeing for parents (adapted from MBSR)	Parent group	8 weeks × 2 h; total 16 h
Lewallen and Neece (2015)#	*N* = 24 mothers	*M* = 3.40 (2.5–5) 67% boys	Non-clinical	Clinical: ASD (83%), other developmental delay (17%)	RCT: 1. MBSR 2. Wait list control	MBSR: MBSR program Control: Nil (offered MBSR after waitlist)	MBSR: Parent group Control: Nil	MBSR: 8 weeks × 2 h + 6 h retreat; total 22 h Control: Nil
Lo et al. (2017a)	*N* = 180 parents (94% mothers)	NR (pre-school age) 77% boys	Non-clinical	Clinical: ASD (57%), developmental delay (28%), ADHD (7%), other diagnosis (8%)	RCT: 1. MP 2. No treatment control	MP: MP adapted from Bögels (2013) and Coatsworth et al. (2014)Control: Nil (mindfulness workshop, after study)	MP: Parent group Control: Nil	MP: 6 weeks × 1.5 h; total 9 h Control: Nil
Lo et al. (2017b)	*N* = 100 parents (96% mothers)	*M* = 6.25 (5–7)83% boys	Non-clinical	Clinical: ADHD	RCT: 1. MP 2. Wait list control	MP: MP adapted from Bögels and Restifo (2014) and Coatsworth et al. (2010)Control: Nil (offered MP after waitlist)	MP: Parent group and separate child mindfulness group Control: Nil	MP: 6 weeks × 1.5 h; total 9 h (for parent groups). 8 weeks × 1 h (for child groups).Control: Nil
Maloney and Altmaier (2007)	*N* = 12 parents (83% mothers) and 12 children	*M* = 3.9 (2.75–6)Gender NR	Non-clinical: participants recently divorced or separated	Non-clinical	Uncontrolled trial: 1. MP	MP (Placone-Willey, 2002)	Parent group	12 weeks; session length NR; total 15 h
Mann et al. (2016)	*N* = 38 parents (95% mothers)	Mean NR (2–6)Gender NR	Non-clinical: history of depression (≥ 3 episodes and in full/ partial remission)	Non-clinical	RCT: 1. MP + usual care 2. Usual care control	MP: MBCT adapted for parents with history of depression Control: usual care	MP: Parent group Control: Nil	MP: 8 weeks, session length and total hours NRControl: Nil
Meppelink et al. (2016)	*N* = 70 parents (93% mothers)	*M* = 8.7 (range NR)57% boys	Non-clinical	Clinical: ASD (29%), parent-child interaction problem (26%), ADHD (24%), AD (3%), ODD (1.5%), adjustment disorder (1.5%), other diagnosis (6%)	Uncontrolled trial: 1. MP	MP (Bögels and Restifo, 2014)	Parent group	8 weeks × 3 h; total 24 h
Neece (2014)	*N* = 46 parents (78% mothers)	*M* = 3.84 (2.5–5)71% boys	Non-clinical	Clinical: ASD	RCT: 1. MBSR 2. Wait list control	MBSR: MBSRControl: Nil (offered MBSR after waitlist)	MBSR: Parent group Control: Nil	MBSR: 8 weeks × 2 h + 6 h retreat; total 22 h Control: Nil
Potharst et al. (2017)	*N* = 37 mothers	*M* = 0.86 (0–1.5) 50% boys	Clinical: mental health disorder (84%) or referral for difficulties related to mothering	Non-clinical: sleeping problems (27%), excessive crying (18%)	Uncontrolled trial: 1. MP	MP adapted for mothers with a baby (Bögels et al., 2014)	Mother/baby group	8 weeks × 2 h; total 16 h
Potharst et al. (2018)^a^ Non-clinical setting	*N* = 98 parents (82% mothers)	*M* = 8.9 (0–35.3) Gender NR	Non-clinical, self-reported parenting stress	Non-clinical	WLC trial: 1. MP	MP shortened for non-clinical context (Bögels and Restifo, 2013)	Parent group	8 weeks × 2 h; total 16 h^b^
Potharst et al. (2018) Clinical setting	*N* = 89 parents (80% mothers)	*M* = 11.7 (2.6–25.4) Gender NR	Non-clinical	Clinical: ADHD (31%), ASD (23%), DICA (10%), AD (5%), PTSD (4%), MD (1%), OCD (1%), ODD (1%), IED (1%), unknown diagnosis (21%)	Uncontrolled trial: 1. MP	MP (Bögels and Restifo, 2013)	Parent group	8 weeks × 3 h + 3 h booster session, 8 weeks post-completion; total 27 h ^c^
Racey et al. (2017)	*N* = 29 parents (97% mothers) and 25 adolescents	*M* = 16.4 (14–18) 0% boys	Non-clinical: 50% parents had history of depression	Clinical: partially recovered from depressive episode	Uncontrolled trial: 1. MBCT	MBCT adapted for parents and youth	Parent group and separate adolescent mindfulness group	8 weeks (for both parent and adolescent groups); session length and total hours NR
Ridderinkhof et al. (2017)	*N* = 74 parents (58% mothers) and 45 adolescents	*M* = 13.03 (8–19) 80% boys	Non-clinical	Clinical: ASD (IQ ≥ 80)	Uncontrolled trial: 1. MP	MP adapted for parents of children with ASD from Bögels and Restifo (2014)	Parent group and separate adolescent mindfulness group	9 weeks × 1.5 h (for both parent and adolescent groups) + 1x joint parent/ adolescent booster session, 9 weeks post-completion; total 15 h
Short et al. (2017)	*N* = 59 mothers	NR (≤3)Gender NR	Clinical: in treatment for opioid and other substance-use disorders	Non-clinical	Uncontrolled trial: 1. MP	MP adapted from MBSR for parents with high rates of trauma	Parent group	12 weeks × 2 h; total 24 h
van de Weijer-Bergsma et al. (2012)	*N* = 11 parents (55% mothers) and 10 adolescents	*M* = 13.4 (11–15) 50% boys	Non-clinical	Clinical: ADHD	Uncontrolled trial: 1. MP	MP (Bögels et al., 2008 and van der Oord et al., 2012)	Parent group and separate adolescent mindfulness group	8 weeks × 1.5 h (for both parent and child groups) + 1x joint parent/ adolescent booster session, 8 weeks post-completion; total ~13 h
van der Oord et al. (2012)	*N* = 22 parents (95% mothers) and 22 children	*M* = 9.55 (8–12) 73% boys	Non-clinical	Clinical: ADHD	WLC trial: 1. MP	MP adapted for parents of children with ADHD from Bögels et al. (2008) and Bögels et al. (2010)	Parent group and separate mindfulness group for children	8 weeks × 1.5 h; total 12 h (for both parent and child groups)
Voos (2017)	*N* = 21 parents (71% mothers)	M = 9.5 (range NR; < 18) 91% boys	Non-clinical	Clinical: ASD	Uncontrolled trial: 1. MP	MP (Bögels and Restifo, 2013)	Parent group	8 weeks × 1.5 h; total 12 h
Xu (2017)#	*N* = 32 parents (90% mothers)	M = 4.68 (2.5–5) 71% boys	Non-clinical	Clinical: ASD (48%), ID or other developmental delay (36%), Down's syndrome (16%)	Uncontrolled trial: 1. MBSR	MBSR	Parent group	8 weeks × 2 h + 6 h retreat; total 22 h
Zhang et al. (2017)	*N* = 11 parents (64% mothers) and 11 children	*M* = 9.5 (8–12) 73% boys	Non-clinical	Clinical: ADHD	Uncontrolled trial: 1. MP	MP (van der Oord et al., 2012; van de Weijer-Bergsma et al., 2012)	Parent group and separate child mindfulness group	8 weeks × 1.5 h; total 12 h (for both parent and child groups)

∧ For both parent and youth clinical status, “Clinical” means that the participating parent or their child were selected for the study based on either a clinical diagnosis, or referral for clinical assistance, for a mental health difficulty. “Non-clinical” means the participating parents, or their child, were not selected for the study based on either a clinical diagnosis or referral for clinical assistance. A non-clinical group of parents or youth may still, therefore, include individuals who meet criteria for a psychiatric or physical health condition; NR, Not reported; MBSR, Mindfulness-Based Stress Reduction (Kabat-Zinn et al., 1992); MBCT, Mindfulness-Based Cognitive Therapy (Segal et al., 2002); MP, mindful parenting; WLC, waitlist controlled; RCT, randomized, controlled trial; ASD, an autism spectrum disorder; ID, an intellectual disability; DD, a depressive disorder; PTSD, post-traumatic stress disorder; ADHD, attention deficit/hyperactivity disorder; PDD, pervasive developmental disorder; ODD, oppositional defiant disorder; CD, conduct disorder; BD, bipolar disorder; BPD, borderline personality disorder; AD, anxiety disorder; LD, learning disorder; OCD, obsessive compulsive disorder; MD, mood disorder; IED, intermittent explosive disorder; DICA, disorder of infancy, childhood or adolescence not otherwise specified; VABS, Vineland Adaptive Behavior Scales (Sparrow et al., 1984);

# Chan and Neece (2018), Lewallen and Neece (2015), and Xu (2017) are included in this table for clarity, however these three studies appear to utilize samples of participants overlapping with Neece (2014);

a Potharst et al. (2018) included two separate streams of participants. One stream attended the intervention in non-clinical settings, the other attended in clinical settings. Study characteristics are reported separately for each setting, given they were independent from each other;

b basic non-clinical program was 8 weeks × 2 h. However, there were 4 locations (A, B, C, and D) and some varied the basic program. B ran 2.5 h sessions, D ran 3 h sessions, and B and D offered a follow-up session;

c *basic clinical program was 8 weeks × 3 h + 3 h booster. This was run at 4 locations (E, F, G, and H). Location E adjusted the session length to 2.5 h*.

Effect sizes reported by the study authors for parenting stress and youth psychological outcomes were also extracted and are included in Tables 2, 3, respectively.

**Table 2:** Reported results of mindfulness intervention, for parenting stress.

**Study**	**Parenting stress measure^**#**^**	**Within group results**		**Between group results**	
		**Pre-post**	**Pre-follow up^**∧**^**	**Pre-post**	**Pre-follow up^**∧**^**
Bazzano et al. (2015)	PSS	NR^a^ (+)	NR^a^ (+)	–	–
Bögels et al. (2014)	PSI, Competence scale	*d* = 0.44 (+)	*d* = 0.47 (+)	–	–
Chaplin et al. (2018)	SIPA subscales:				
	Parent Life Restrictions	–	–	*d* = 0.53 (+)	–
	Parent Incompetence/Guilt	–	–	*d* = −0.14	–
	Relationship with Partner	–	–	*d* = 0.59 (+)	–
Corthorn (2018)	PSI–SF	–	–	NR (+)	*d* = 0.66 (+)
De Bruin et al. (2015)	PSI	*d* = 0.21 (+)	*d* = −0.01	–	–
Eames et al. (2015)	PSI–SF	*g* = 0.81^b^	–	–	–
Ferraioli and Harris (2013)	PSI–SF	*d* = 2.03 (+)	*d* = 1.01	*d* = 1.59 (+)	*d* = 0.63
Haydicky et al. (2015)	SIPA	NR	*d* = 0.81 (+)	–	–
Jones et al. (2018)	QRS-PFP	*d* = −0.12	–	–	–
Lo et al. (2017a)	PSI-SF	–	–	*d* = 0.34 (+)	–
Lo et al. (2017b)	PSI-SF	–	–	*d* = 0.19 (+)	–
	HRV Low frequency^c^	–	–	*d* = 0.00	–
Maloney and Altmaier (2007)	PSI-SF	*d* = 0.26	–	–	–
Mann et al. (2016)	PSI-SF	–	–	*d* = 0.40 (4 mo.)	*d* = 0.40 (9 mo.)
Neece (2014)	PSI-SF, Parental Distress scale	*d =* 0.70 (+)^d^	–	*d* = 0.70 (+)	–
Potharst et al. (2017)	PSI, modified version	*d* = 0.25	*d* = 0.44 (+); *d* = 0.53 (+) (1 yr.)	–	–
Potharst et al. (2018)	OBVL	*d* = 0.37 (+)	*d* = 0.67 (+)	–	–
Ridderinkhof et al. (2017)	PSI, Competence scale	*d* = 0.21 (+)	*d* = 0.39 (+); *d* = 0.28 (+) (1 yr.)	–	–
Short et al. (2017)	PSI-SF	*d* = 0.04	–	–	–
van de Weijer-Bergsma et al. (2012)	PSI–SF	*d* = −0.50^M^; *d* = 0.70^F^ (+)	*d* = −0.20^M^; *d* = 1.1^F^ (+)	–	–
van der Oord et al. (2012)	PSI-SF	NR (ns)	*d* = 0.57 (+)	–	–
Voos (2017)	PSI	NR	*d* = 0.94 (+)	–	–
Zhang et al. (2017)	PSI-SF	*d* = −0.18 (+)	–	–	–

# *= all parenting stress effects are based upon the reports of the parent/s who attended the intervention, and therefore combine mother and father reports, except in the case of van de Weijer-Bergsma et al. (2012) which reports mother and father results separately; ^∧^ = 8 week follow up, unless otherwise indicated; (+) indicates effect size is significant (as reported by the relevant study author/s), p < .05. For within-group results, effect size is reported as a positive number if there was improvement in the outcome, and as a negative number if there was a deterioration. For between-group results, effect size is reported as a positive number if the outcome improved more in the mindfulness group than the control group; NR = not reported; ns = not significant; ^a^ = d not reported, but % change reported as significant; ^b^ g = Hedges' glass; ^c^ = only low frequency heart rate variability (HRV) is included, as the effect for high frequency HRV was reported only as non-significant; ^d^ = the within-group parenting stress effect is reported in Xu (2017); ^M^ = mother; ^F^ = father; PSS = Parental Stress Scale (Berry and Jones, 1995); PSI = Parenting Stress Index (Abidin, 1983); PSI-SF = Parenting Stress Index, Short Form (Abidin, 1995); SIPA = Stress Index for Parents of Adolescents (Sheras et al., 1998); QRS-PFP = Questionnaire on Resources and Stress Short Form – Parent and Family Problems subscale (Friedrich et al., 1983); OBVL = Opvoedingsbelastingvragenlijst, Veerman et al. (2014), a Dutch parenting stress questionnaire*.

**Table 3:** Reported results of mindfulness intervention, for youth psychological outcomes.

**Study**	**Outcomes**	**Measure**	**Reporter**	**Within group results**		**Between group results (Pre-post)**
				**Pre-post**	**Pre-follow up^**∧**^**	
Bögels et al. (2008)	Mindfulness	MAAS	Youth	*d* = 0.50 (+)	*d* = 0.50 (+)	–
	Internalizing outcomes:					
	Internalizing problems	YSR	Youth	*d* = 0.50	*d* = 0.50	–
		CBCL	Parent	*d* = −0.10	*d* = 0.30	–
	Happiness	SHS	Youth	*d* = 0.60 (+)	*d* = 0.60 (+)	–
	Externalizing outcomes:					
	Externalizing problems	YSR	Youth	*d* = 1.10 (+)	*d* = 1.20 (+)	–
		CBCL	Parent	*d* = 0.30	*d* = 0.40	–
	Self-control	SCRS	Youth	*d* = 0.80 (+)	*d* = 0.60 (+)	–
	Cognitive outcomes:					
	Thought problems	YSR	Youth	*d* = 0.40	*d* = 0.30	–
		CBCL	Parent	*d* = 0.00	*d* = 0.10	–
	Attention problems	YSR	Youth	*d* = 1.00 (+)	*d* = 0.90 (+)	–
		CBCL	Parent	*d* = 0.30	*d* = 0.50	
	Sustained attention	D2 Test of Attention	Youth	*d* = 0.60 (+)	*d* = 1.10 (+)	
	Social outcomes:					
	Social problems	YSR	Youth	*d* = 0.60 (+)	*d* = 0.50 (+)	–
		CBCL	Parent	*d* = 0.20	*d* = 0.30	–
	Social behavior	CSBQ	Parent	*d* = −0.10	*d* = 0.40	–
Bögels et al. (2014)	Internalizing outcomes:					
	Internalizing problems	CBCL	Parent	*d* = 0.45 (+)	*d* = 0.47 (+)	–
	Externalizing outcomes:					
	Externalizing problems	CBCL	Parent	*d* = 0.31 (+)	*d* = 0.37 (+)	–
De Bruin et al. (2015)	Mindfulness	MAAS – A	Youth	*d* = −0.26	*d* = −0.02	^−^
	Internalizing outcomes:					
	Worry	PSWQ	Youth	*d* = −0.04	*d* = 0.28	–
	Rumination	RRS	Youth	*d* = 0.34	*d* = 0.92 (+)	–
	Well-being	WHO-5	Youth	*d* = 0.55 (+)	*d* = 0.63 (+)	–
	Externalizing outcomes:					
	Autism core symptoms	AQ	Youth	*d* = −0.04	*d* = 0.06	–
			Parent	*d* = 0.09	*d* = −0.15	
	Social outcomes:					
	Social responsiveness	SRS	Parent	*d* = −0.01	*d* = 0.33	–
Haydicky et al. (2015)^a^	Internalizing outcomes:					
	Internalizing problems	RCADS	Youth	*d* = 0.26	*d* = 1.01 (+)	–
			Parent	NR	*d* = 0.49	
	Anxiety	RCADS	Youth	*d* = 0.25	*d* = 1.02 (+)	–
			Parent	NR	*d* = 0.37	
	Depression	RCADS	Youth	*d* = 0.38	*d* = 0.64 (+)	–
			Parent	NR	*d* = 0.55	
	Externalizing outcomes:					
	ODD	Conners	Youth	*d* = −0.45	*d* = 0.21	–
			Parent	NR	*d* = 0.45	
	CD	Conners	Youth	NR	*d* = 0.46	–
			Parent	*d* = 0.70 (+)	*d* = 0.32	
	Hyperactivity/impulsivity	Conners	Youth	NR	*d* = 0.16	–
			Parent	NR	*d* = 0.41	
	Cognitive outcomes:					
	Inattention	Conners	Youth	NR	*d* = 0.12	–
			Parent	*d* = 0.62	*d* = 0.20	
	Learning problems	Conners	Youth	NR	*d* = −0.64	–
			Parent	*d* = 0.46	*d* = 0.29	
	Executive function	Conners	Parent	*d* = 0.36	*d* = 0.24	–
	Social outcomes:					
	Peer relations	Conners	Parent	*d* = 1.07 (+)	*d* = 0.02	–
	Family relations	Conners	Youth	*d* = −0.34	*d* = 0.31	–
Jones et al. (2018)	Externalizing outcomes:					
	Behavior problems	SDQ	Parent	*d* = −0.14	–	–
	Social outcomes:					
	Prosocial behavior	SDQ	Parent	*d* = 0.04	–	–
Lo et al. (2017a)	Externalizing outcomes:					
	Behavior problems	ECBI	Parent	–	–	NR (ns)
	Behavior severity	ECBI	Parent	–	–	NR (ns)
Lo et al. (2017b)	Internalizing outcomes:					
	Internalizing problems	CBCL	Parent	–	–	*d* = 0.46 (+)
	Externalizing outcomes:					
	Externalizing problems	CBCL	Parent	–	–	*d* = 0.29 (+)
	ADHD symptoms	SWAN	Parent	–	–	*d* = 0.63 (+)
	Executive function^b^	CANT Conflict monitoring	Youth	–	–	*d* = 0.41 (+)
Mann et al. (2016)	Externalizing outcomes:					
	Behavior problems	SDQ	Parent	–	–	*d* = 0.60 (+) (4 mo.)
Meppelink et al. (2016)	Internalizing outcomes:					
	Internalizing problems	CBCL	Parent	*d* = 0.34 (+)	*d* = 0.31 (+)	–
	Externalizing outcomes:					
	Externalizing problems	CBCL	Parent	*d* = 0.22 (+)	*d* = 0.37 (+)	–
	Cognitive outcomes:					
	Attention problems	CBCL	Parent	*d* = 0.26 (+)	*d* = 0.42 (+)	–
Neece (2014) [including Lewallen and Neece (2015); Xu (2017); Chan and Neece (2018)]	Internalizing outcomes:					
	Internalizing problems	CBCL	Parent	–	–	*d* = −0.13
	Emotional reactivity	CBCL	Parent	–	–	*d* = −0.31
	Anxious/depressed	CBCL	Parent	–	–	*d* = −0.25
	Somatic complaints	CBCL	Parent	–	–	*d* = 0.24
	Withdrawn/depressed	CBCL	Parent	–	–	*d* = −0.04
	Sleep problems	CBCL	Parent	–	–	*d* = 0.28
	DSM Affective problems	CBCL	Parent	–	–	*d* = 0.57
	DSM Anxiety problems	CBCL	Parent	–	–	*d* = −0.20
	Emotion dysregulation^c^	DCS	Observer	β = 0.27, sr^2^ = 0.06	–	–
	Emotion regulation^d^	ERC	Parent	*d* = 0.12	–	–
	Externalizing outcomes:					
	Externalizing problems	CBCL	Parent	–	–	*d* = 0.45
	Aggressive behavior	CBCL	Parent	–	–	*d* = 0.30
	DSM ADHD problems	CBCL	Parent	–	–	*d* = 0.85 (+)
	DSM ODD	CBCL	Parent	–	–	*d* = 0.20
	Cognitive outcomes:					
	Attention problems	CBCL	Parent	–	–	*d* = 0.71
	DSM Developmental problems	CBCL	Parent	–	–	*d* = 0.17
	Social outcomes^e^:	SSIS				
	Self-control		Parent	*d* = 0.54 (+)	–	–
			Secondary Informant	*d* = 0.36 (+)		
			Teacher	*d* = 0.59 (+)		
	Communication		Parent	*d* = 0.03	–	–
			Secondary Informant	*d* = 0.10		
			Teacher	*d* = 0.75 (+)		
	Cooperation		Parent	*d* = −0.03	–	–
			Secondary Informant	*d* = 0.12		
			Teacher	*d* = 0.83 (+)		
	Assertion		Parent	*d* = −0.24	–	–
			Secondary Informant	*d* = 0.74 (+)		
			Teacher	*d* = 0.48 (+)		
	Responsibility		Parent	*d* = 0.18	–	–
			Secondary Informant	*d* = 0.19		
			Teacher	*d* = 0.58 (+)		
	Empathy		Parent	*d* = 0.61 (+)	–	–
			Secondary Informant	*d* = 0.27		
			Teacher	*d* = 0.58 (+)		
	Engagement		Parent	*d* = 0.61 (+)	–	–
			Secondary Informant	*d* = 0.19		
			Teacher	*d* = 0.82 (+)		
Potharst et al. (2017)	Internalizing outcomes:					
	Positive affect	IBQ-R	Parent	*d* = 0.48 (+)	*d* = 0.51 (+)	–
	Regulating	IBQ-R	Parent	*d* = 0.35	*d* = 0.06	–
	Negative emotionality	IBQ-R	Parent	*d* = 0.25	*d* = 0.19	–
Potharst et al. (2018)	Internalizing outcomes:					
	Well-being	WHO-5	Parent	*d* = 0.30 (+)	*d* = 0.11	–
	Externalizing outcomes:					
	Behavior problems	SDQ	Parent	*d* = 0.61 (+)	*d* = 0.41 (+)	–
Racey et al. (2017)	Internalizing outcomes:					
	Depression	BDI-II	Youth	NR (+)^f^	–	–
	Rumination	RRS	Youth	NR (+)^f^	–	–
	Self–compassion	SCS	Youth	NR (+)^f^	–	–
	De-centring	EQD	Youth	NR (+)^f^	–	–
Ridderinkhof et al. (2017)	Mindfulness	CAMM^g^	Youth	*d* = 0.02	*d* = 0.37; *d* = 0.01 (1 yr.)	
	Internalizing outcomes:					
	Internalizing problems	YSR^g^	Youth	*d* = 0.13	*d* = 0.50; *d* = 0.59 (1 yr.)	–
		CBCL	Parent	*d* = 0.35 (+)	*d* = 0.38 (+); *d* = 0.63 (+) (1 yr.)	–
	Rumination	RRS^g^	Youth	*d* = 0.44 (+)	*d* = 0.71 (+); *d* = −0.27 (1 yr.)	–
	Stress	CSQ-CA	Youth	*d* = 0.20	*d* = 0.63 (+); *d* = 0.25 (1 yr.)	–
	Sleep problems	CSRQ	Youth	*d* = 0.06	*d* = 0.28; *d* = 0.12 (1 yr.)	–
	Well-being	WHO-5	Youth	*d* = 0.35	*d* = 0.40; *d* = 0.46 (+) (1 yr.)	–
	Externalizing outcomes:					
	Externalizing problems	YSR^g^	Youth	*d* = 0.20	*d* = 0.56 (+); *d* = 0.61 (+) (1 yr.)	–
		CBCL	Parent	d = 0.21 (+)	d = 0.43 (+); d = 0.42 (+) (1 yr.)	–
	Cognitive outcomes:
	Attention problems	YSR^g^	Youth	*d* = 0.22	*d* = 0.57 (+); *d* = 0.68 (+) (1 yr.)	–
		CBCL	Parent	*d* = 0.32 (+)	*d* = 0.44 (+); *d* = 0.58 (+) (1 yr.)	–
	Social outcomes:					
	Social responsiveness	SRS	Parent	*d* = 0.32 (+)	*d* = 0.33 (+); *d* = 0.51 (+) (1 yr.)	–
van der Oord et al. (2012)	Externalizing outcomes:					
	Inattention	DBDRS	Parent	*d* = 0.80 (+)	*d* = 0.80 (+)	–
			Teacher	NR (ns)	NR (ns)	
	Hyperactivity	DBDRS	Parent	*d* = 0.56 (+)	*d* = 0.59 (+)	–
			Teacher	NR (ns)	NR (ns)	
	ODD	DBDRS	Parent	NR (ns)	NR (ns)	–
			Teacher	NR (ns)	NR (ns)	
van de Weijer-Bergsma et al. (2012)	Mindfulness	MAAS	Youth	*d* = 0.10	*d* = −0.10; *d* = 0.50 (16 wks.)	–
	Internalizing outcomes:					
	Internalizing problems	YSR	Youth	*d* = 0.10	*d* = 0.20; *d* = 0.70 (16 wks.)	–
		CBCL	Mother	*d* = 0.10	*d* = 0.00	–
			Father	*d* = 0.40	*d* = 0.50	
			Teacher	*d* = 0.20	–	
	Fatigue	FFS	Youth	*d* = 0.00	*d* = 0.20; *d* = −0.10 (16 wks.)	–
	Happiness	SHS	Youth	*d* = −0.50	*d* = −0.40; *d* = −0.20 (16 wks.)	–
	Externalizing outcomes:					
	Externalizing problems	YSR	Youth	*d* = −0.10	*d* = 0.50; *d* = 0.90 (16 wks.)	–
		CBCL	Mother	*d* = −0.21	*d* = 0.10	–
			Father	*d* = 0.20 (+)	*d* = 0.30 (+)	
			Teacher	*d* = 0.20	–	
	Cognitive outcomes:					
	Attention problems	YSR	Youth	*d* = 0.50	*d* = 0.90 (+); *d* = 1.0 (16 wks.)	–
		CBCL	Mother	*d* = 0.10	*d* = 0.30	–
			Father	*d* = 0.60	*d* = 1.50 (+)	
			Teacher	*d* = 0.30	–	
	Metacognitive problems	BRIEF	Mother	*d* = −0.30	*d* = 0.00	–
			Father	*d* = 1.00	*d* = 1.80 (+)	
			Teacher	*d* = 0.20	–	
	Behavior regulation problems	BRIEF	Mother	*d* = −0.20	*d* = 0.10	–
			Father	*d* = 0.10	*d* = 0.60 (+)	
			Teacher	*d* = −0.50	–	
	Reaction time	ANT	Youth	*d* = −0.20	*d* = −0.10; *d* = −0.70 (16 wks.)	
	Sustained attention^h^	ANT	Youth	*d* = 0.20 to *d* = 0.40	*d* = 0.80 (+); *d* = 0.40 to *d* = 0.50 (16 wks.)	
	Impulsivity^i^	ANT	Youth	*d* = 0.00 to *d* = 0.50 (+)	*d* = 0.30 to *d* = 0.70; *d* = 0.10 to *d* = 0.70 (16 wks.)	
Zhang et al. (2017)	Externalizing outcomes:					
	Behavior problems	ECBI	Parent	*d* = 0.25	–	–
	Behavior severity	ECBI	Parent	*d* = 0.36 (+)	–	–
	Cognitive outcomes:					
	Metacognitive problems	BRIEF	Parent	*d* = 0.00	–	–
	Behavior regulation problems	BRIEF	Parent	*d* = 0.01	–	–
	Sustained attention^j^	Tea–CH	Youth	*d* = −0.24 to *d* = 0.76	–	–
	Selective/focussed attention^k^	Tea-CH	Youth	*d* = 0.80 to *d* = 1.53 (+)	–	–
	Attentional control/switching^l^	Tea-CH	Youth	*d* = −0.16 to *d* = 0.81	–	–
	Inattention^m^	CCPT	Youth	*d* = −0.43 to *d* = 2.29 (+)	–	–
	Impulsivity^n^	CCPT	Youth	*d* = −0.73 to *d* = 0.81	–	–
	Vigilance°	CCPT	Youth	*d* = −0.13	–	–
	Sustained attention^p^	CCPT	Youth	*d* = 0.28	–	–

For within-group results, effect size is reported as a positive number if there was an improvement in the outcome, and as a negative number if there was a deterioration. For between-group results, effect size is reported as a positive number if the outcome improved more in the mindfulness group than the control group; + indicates effect size is significant, p < 0.05;

∧ , 8 week follow up, unless otherwise indicated; NR, not reported by study authors; ns, not significant;

a the follow-up effects reported by Haydicky et al. (2015) are post-follow up;

b only the conflict monitoring effect is included, as effects for alerting, orienting, response time, and accuracy were reported only as non-significant;

c Emotion dysregulation effect is reported in Chan and Neece (2018);

d Emotion regulation is reported in Xu (2017);

e Social skills are reported in Lewallen and Neece (2015);

f d not reported, but mean change reported as significant;

g these measures were only completed by adolescents ≥11years;

h Sustained attention measured by “misses” measures of Amsterdam Neuropsychological Tasks (ANT; De Sonneville, 1999);

i Impulsivity measured by “false alarms” measures of ANT;

j Sustained attention measured by Score!, Sky Search DT, Walk Do Not Walk, and Code Transmission subtests of the Test of Everyday Attention for Children (Tea-CH; Manly et al., 2001);

k Selective/focussed attention measured by Sky Search and Map Mission subtests of Tea-CH;

l Attentional control/switching measured by Creature Counting and Opposite Worlds subtests of Tea-CH;

m Inattention measured by detectability, omissions, commissions, Hit reaction time (HRT) statistics, and variability measures in Conners' Continuous Performance Test, 3rd edition (CCPT; Conners, 2015);

n Impulsivity measured by commissions, perseverations, and HRT measures of CCPT; °Vigilance measured by HRT block change measure of CCPT;

p *Sustained attention measured by HRT block change measure of CCPT; MAAS, Mindful Attention and Awareness Scale (Brown and Ryan, 2003); YSR, Youth Self-Report (Achenbach, 1991a); CBCL, Child Behavior Checklist (Achenbach, 1991b); SHS, Subjective Happiness Scale (Lyubomirsky and Lepper, 1999); SCRS, Self Control Rating Scale (Kendall, 1979); CSBQ, Children's Social Behavior Questionnaire (Luteijn et al., 2000); MAAS-A, Mindful Attention and Awareness Scale–Adolescent (Brown et al., 2011); PSWQ, Penn State Worry Questionnaire (Meyer et al., 1990); Ruminative Response Scale (Nolen-Hoeksema, 2000); WHO-5, World Health Organization-Five Wellbeing Index (Bech et al., 2003); SRS, Social Responsiveness Scale (Constantino and Gruber, 2005); AQ, Autism Questionnaire (Auyeung et al., 2008); RCADS, Revised Child Anxiety and Depression Scale (Chorpita et al., 2000); Conners, Conners 3rd Edition (Conners, 2008); SDQ, Strengths and Difficulties Questionnaire (Goodman, 1997); ECBI, Eyberg Child Behavior Inventory (Robinson et al., 1980); SWAN, Strengths and Weaknesses of ADHD Symptoms and Normal Behaviors Rating Scale (Swanson et al., 2012); CANT, Child Attention Network Test (Posner and Petersen, 1990); DCS, Dysregulation Coding System (Hoffman et al., 2006); ERC, Emotion Regulation Checklist (Shields and Cicchetti, 1997); SSIS, Social Skills Improvement System (Gresham and Elliott, 2008); IBQ-R, Infant Behavior Questionnaire-Revised, Very Short Form (Putnam et al., 2014); BDI-II, Beck Depression Inventory (Beck et al., 1996); SCS, Self Compassion Scale (Neff, 2015); EQD, Experiences Questionnaire (Fresco et al., 2007), Decentring subscale; CAMM, Children's Acceptance and Awareness Measure (De Bruin et al., 2013); CSQ-CA, Chronic Stress Questionnaire for Children and Adolescents (De Bruin et al., 2017); CSRQ, Chronic Sleep Reduction Questionnaire (Meijer, 2008); DBDRS, Disruptive Behavior Disorder Rating Scale (Pelham et al., 1992); FFS, Flinders Fatigue Scale (Gradisar et al., 2007); BRIEF, Behavior Rating Inventory of Executive Function (Goia et al., 2000)*.

Quantitative data needed for calculation of effect sizes in the meta-analysis were also extracted. Where a study did not report the data required for calculation of effect sizes, they were requested by email from the corresponding author of the study. If no response was received, the study was included in the systematic review (in Tables 1–3), but not included in the quantitative analyses.

### Data Analysis

The meta-analysis was conducted using the Comprehensive Meta-Analysis program, version 3.0 (CMA). Two types of summary effect were calculated, using means and standard deviations whenever these were available, and statistics such as *t* and *p* when they were not. For studies reporting pre- and post-intervention outcome data, we calculated Hedges' *g* within-group effect sizes. For studies comparing outcomes of mindfulness and control groups, we calculated Hedges' *g* between-group differences in effect size. Hedges' *g* is a weighted mean effect size that corrects for potential bias due to small sample sizes (Hedges and Olkin, 1985). Cohen's guidelines that an effect size of 0.20 is small, 0.50 is moderate and 0.80 is large (Cohen, 1988) may be applied to both Cohen's *d* and Hedges' *g* effect sizes. For all analyses, a correlation of *r* = 0.70 was assumed between pre- and post-intervention measures (Rosenthal, 1993). Random-effects models were used for main effects analyses, to reflect the assumption that the true effect size would vary from study to study because study participants were drawn from different populations. Each summary effect reported in this paper is therefore an estimate of the mean of a distribution of true effects (Borenstein et al., 2009). Heterogeneity amongst studies in each main-effect analysis was assessed using the *Q* and *I*^2^ statistics. *Q* reflects the distance of each study from the summary effect. A significant *Q*-statistic indicates variance in true effects, rather than variance due only to random sampling error (Borenstein et al., 2009). *I*^2^ reflects the proportion of observed variance in effects that is due to heterogeneity, or variance in true effects (Higgins et al., 2003). Higgins et al. suggest that *I*^2^ values of 25, 50, and 75% indicate low, moderate, and high heterogeneity, respectively.

Several methodological issues arose in connection with the calculation of the summary effect size for parenting stress. All studies except one reported either a total parenting stress score or the score from a single parenting stress subscale. A parenting stress effect size was therefore calculated for each of these studies, using the single reported outcome score. However, Chaplin et al. (2018) reported separate data for three subscales of the Stress Index for Parents of Adolescents (SIPA; Sheras et al., 1998). Rather than including each of these three subscales as independent effects in the meta-analysis, the procedure described by Borenstein et al. (2009) was followed to create a single, composite effect for this study. Using a single effect ensures that additional weight is not given to this study, as would be the case if the subscales were treated as independent of each other. It also ensures that the precision of the summary effect is not over-estimated due to the positive correlations between each subscale (Borenstein et al., 2009). Under this procedure, the effects for each subscale were averaged to give a composite parenting stress effect size. To calculate the variance of the composite effect, a correlation between the subscales of *r* = 0.55 was used, based on the reported correlations between the three relevant subscales of *r* = 0.52–0.57 (Sheras et al., 1998). A similar issue arose in relation to the parenting stress reporter. Although the majority of studies presented data for a single parenting stress reporter, van de Weijer-Bergsma et al. (2012) reported separate data for mothers and fathers. As mothers and fathers were reporting their levels of stress in respect of the same adolescent, the mother and father effects were not independent. Accordingly, a composite mother/father effect size was calculated following the procedure described above, using a correlation between the two outcomes of *r* = 0.60. This *r*-value was chosen using the correlations between mother- and father-reports of child anxiety (*r* = 0.68) and parental rearing (*r*s between 0.39 and 0.49) reported in Bögels and van Melick (2004), as a guide. Finally, Potharst et al. (2018) reported data separately for parents participating in clinical and non-clinical settings. The effects reported for these two settings have been included separately in all analyses, as if they were data from two separate studies, because they are based on reports from independent groups of parents participating in independent settings.

Due to the limited number of studies reporting on specific youth psychological outcomes, a detailed quantitative analysis was not conducted in respect of each youth outcome covered by the reviewed studies. Instead, specific outcomes were grouped into internalizing, externalizing, cognitive, and social domains, as the reported outcomes all fell within one of these four domains of functioning. In addition, to provide a large enough pool of effects for moderator analyses to be conducted, a new “overall youth outcomes” variable was created. This variable was created by first calculating effect sizes for youth outcomes reported by parents and then calculating a single, composite parent-reported effect size for each study using the Borenstein et al. (2009) procedure described above, assuming a correlation between the outcomes within each study of *r* = 0.60. In studies reporting a broadband scale for youth outcomes (for example, “Internalizing problems”), the effect for the broadband scale was used in the calculation of the overall youth outcomes summary effect size. Where a study also reported data for the specific scales making up that broadband scale, specific scale effects were not included. In studies where no broadband scale was used, but more than one youth psychological outcome was reported (for example, anxiety and depression), then these were combined to form a composite effect. For studies reporting data for only one relevant youth outcome, then the effect size for that outcome was used for that study. For the two studies that reported separate youth outcome data for two parents or a parent and another family caregiver (van de Weijer-Bergsma et al., 2012; Lewallen and Neece, 2015), a composite parent-reported effect size was calculated using a correlation of *r* = 0.60 between the two parent or caregiver outcomes. The same two studies also included data from tutor reports on some outcomes. However, for consistency with the other studies, the tutor-reported data was not included in the calculation of the youth outcomes effect for those two studies. Data from youth-reported and objective tests of youth outcomes were also not used, as most studies did not include these data. The single youth outcome effect size for each study was then combined with the others to generate a summary, parent-reported overall youth outcome effect size.

Exploratory moderator analyses were conducted in relation to both parenting stress and overall youth outcomes. For potential categorical moderators, a mixed effects model was used (random-effects within subgroups and fixed-effects across subgroups). The variance of true effect sizes across studies (*T*^2^) was estimated by pooling within-group estimates of *T*^2^ for each subgroup and applying the common estimate to all studies. This method of estimating *T*^2^ is recommended by Borenstein et al. (2009) to increase the accuracy of the estimate, when the number of studies within any subgroup is low. Categorical moderators were tested only when there were four or more studies per subgroup (Fu et al., 2011). To test significance, the *Q* statistic was calculated between subgroups (*Q*_B_). Random-effects meta-regression analyses were used to investigate the relationship between parent or youth outcomes and potential continuous moderators.

### Risk of Bias in Individual Studies

A risk of bias assessment was conducted for each included study. Bias is defined as the tendency for study results to vary from those that would have been obtained from a well-designed and run RCT on the same participant group (Sterne et al., 2016). The domains assessed for potential bias were confounding (for non-randomized studies only), selection, misclassification, performance, attrition, detection and reporting bias. For RCTs, the Cochrane Risk of Bias tool for Randomized Controlled Trials (Higgins et al., 2011) was used to assess selection bias. However, for all other domains, the Cochrane Risk of Bias in Non-randomized Studies of Interventions (ROBINS-I) tool (Sterne et al., 2016) was used, as that tool appeared more suited to assessing studies of psychological interventions where blinding of participants, researchers and outcome assessments are not possible. For the non-randomized studies, the ROBINS-I tool was used to assess all domains. All included studies were assessed for potential bias independently by both the first author and the graduate student who assisted with study selection. There was 94% agreement in bias ratings, with differences resolved by discussion.

## Results

### Study Selection

**Figure 1:**
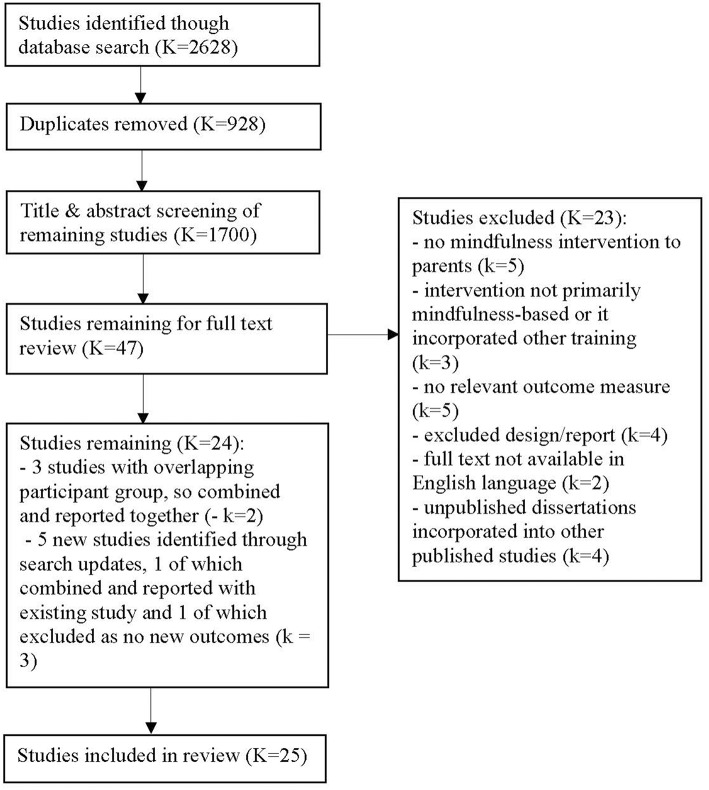
Flow diagram showing process of study selection.

Figure 1 shows the process of study selection and exclusion. The database searches identified 2,628 studies, 928 of which were duplicates. Forty-seven studies were retained after the title and abstract screening. Twenty-three of these studies were excluded based on the full text review, for the reasons set out in Figure 1. Of the 24 retained studies, three studies (Neece, 2014; Lewallen and Neece, 2015; Xu, 2017) appeared to be reporting data from an overlapping participant group. Confirmation was sought by email from the corresponding author but was not received. Lewallen and Neece (2015) and Xu (2017) reported on relevant outcomes that were not included in Neece (2014), but the outcome data for these two studies are reported in Table 3 under Neece (2014), to reflect the apparent non-independence of the outcomes reported in these two studies. When the initial search conducted in August 2018 was updated in October 2018, five additional studies were identified by the first author. Two of these, Chan and Neece (2018) and Neece et al. (2018), also appeared to report data from a group of participants overlapping with those used in Neece (2014). As these two new studies and Neece (2014) all reported on parenting stress, the parenting stress outcomes from Chan and Neece (2018) and Neece et al. (2018) were not included in this review. The child outcome reported by Chan and Neece (2018) was not included in Neece (2014), so this child outcome is reported in Table 3, also under Neece (2014). However, the child outcomes reported in Neece et al. (2018) were also reported in Neece (2014), so this study was not included in this review. Accordingly, 25 independent studies are included in this review.

### Study Characteristics

Twenty-five independent studies reported on the effects of a mindfulness intervention for parents. Eighteen studies delivered mindful parenting interventions, five studies delivered MBSR or Mindfulness-based Cognitive Therapy (MBCT) interventions specifically adapted for parents, and four studies (which appeared to use overlapping participant groups) delivered MBSR to parents. Where adaptations were made to standard MBSR or MBCT programs to reflect the fact that the participants were parents, these adaptations were minor. For example, trainers encouraged participants to reflect on how key concepts of mindfulness, such as acceptance and non-reactivity, might apply to their interactions with their children.

All studies delivered the intervention in a group format. Sixteen studies delivered the intervention to parents (including one mother/infant group), while nine delivered parallel mindfulness training to both parents and their children (parents and children in separate groups). In all studies, the majority of participating parents (between 55 and 100%) were mothers. In relation to parental mental health, four studies involved parents referred for mental health treatment for their own mental health condition or parenting difficulties, while another six studies involved parents identified as being vulnerable to mental health difficulties due to socio-demographic factors or past psychiatric history, or who self-reported experiencing parenting stress. The remaining studies did not report on parental mental health status. In relation to youth mental health, the children of participating parents were identified as having mental health diagnoses or difficulties in 20 of the 25 studies. The mean age of children of participating parents ranged from 0.86 to 16.4 years, and 16 studies involved parents with children whose mean age was <12 years.

Sample sizes ranged from 11 to 180 participants. Of the 25 independent studies, 18 utilized a single group design and seven used a control group. Of the controlled trials, six were RCTs. Two RCTs used an active control group (skills-based parent training and parent education), while the remainder used passive controls such as waitlist or usual care groups. Individual session length ranged from 1.5 h (ten studies) to 3 h (three studies). Eight of the ten studies that delivered parallel parent and child interventions used the shorter 1.5 h sessions. The interventions were delivered over 6–12 weeks, and involved total hours of training between 9 and 27 h.

### Parenting Stress

#### Within-Group Differences

**Figure 2:**
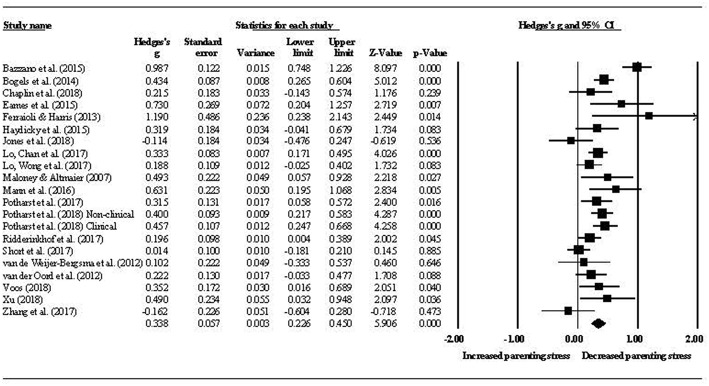
Pre- to post-intervention change in parenting stress.

Nineteen studies reported data enabling a quantitative analysis of within-group parenting stress. Figure 2 shows the effect sizes for pre- to post-intervention change in parenting stress, with a summary Hedges' *g* = 0.34 (*p* < 0.001, 95% CI [0.23–0.45]). Heterogeneity was moderate to high (*Q* = 66.96, *p* = < 0.001, *I*^2^ = 70%). Figure 2 reports composite mother/father data for all studies where mothers and fathers participated. In the one study that reported mother and father outcomes separately, the authors found a significant, moderate to large reduction in parenting stress for fathers and a moderate but insignificant increase for mothers (van de Weijer-Bergsma et al., 2012). At first follow-up, which was generally 2 months post-intervention, the summary effect size for change in parenting stress was *g* = 0.53 (*p* < 0.001, 95% CI [0.45–0.61]) and heterogeneity was low (*Q* = 6.62, *p* = 0.76, *I*^2^ = 0%). The difference between pre-post and pre-follow up effect sizes was significant (*Q*_B_ = 7.32, *df* = 1, *p* = 0.007). Two studies also reported a 1-year post-intervention follow up. While no quantitative analysis was conducted for this time-point, the reported small to moderate reductions in parenting stress from pre-intervention remained significant [*d* = 0.53 in Potharst et al. (2017) and *d* = 0.28 in Ridderinkhof et al. (2017)].

Moderator analyses were conducted in relation to youth clinical status (clinical vs. non-clinical), youth age (child under 12 years vs. adolescent 12 years and over), and intervention groups (parent only mindfulness group vs. parallel parent and youth mindfulness groups). There were insufficient studies to conduct this analysis in respect of parent clinical status. No significant difference was found between the parenting stress effect sizes for parents attending a mindfulness program based on youth clinical status (*g* = 0.33, *p* < 0.001, 95% CI [0.19–0.48] for clinical youth and *g* = 0.35, *p* < 0.001, 95% CI [0.16–0.53] for non-clinical youth; *Q*_B_ = 0.01, *df* = 1, *p* = 0.906). Similarly, there was no difference in effects between parents of children (*g* = 0.31, *p* < 0.001, 95% CI [0.21–0.42]) and adolescents (*g* = 0.21, *p* = 0.005, 95% CI [0.06–0.35]) (*Q*_B_ = 1.33, *df* = 1, *p* = 0.248). However, the effect size for studies using parent-only intervention groups (*g* = 0.35, *p* < 0.001, 95% CI [0.24–0.46]) was greater than that for studies using parallel intervention groups (*g* = 0.18, *p* = 0.001, 95% CI [0.07–0.29]) (*Q*_B_ = 4.37, *df* = 1, *p* = 0.036). A meta-regression of total intervention hours on parenting stress effect size provided no evidence of a dose-response relationship between total hours spent in the mindfulness intervention and parenting stress (β = 0.01, *SE* = 0.01, *p* = 0.26).

Parenting stress was assessed by all studies as an outcome variable rather than as a potential mediator in the relationship between mindfulness in parenting and youth outcomes. One study (Haydicky et al., 2015) examined the direction of relationship between mindful parenting and parenting stress, by using cross-lagged panel correlations. Pre-test mindful parenting scores were significantly negatively correlated with post-test parenting stress [*r*_(14)_ = −0.52, *p* = 0.02], but pre-test parenting stress was not significantly correlated with post-test mindful parenting [*r*_(14)_ = −0.13, *p* = 0.311].

#### Between-Group Differences

Five studies reported data enabling a comparison of post-intervention differences in parenting stress between mindfulness and control groups. The summary effect for the difference between these two groups indicated that the mindfulness groups experienced larger reductions in parenting stress than the control groups. This difference was of a small to moderate size (*g* = 0.44, *p* = 0.005, 95% CI [0.13–0.74]), with moderate heterogeneity (*Q* = 8.11, *p* = 0.087, *I*^2^ = 51%). Of these controlled studies, two compared a mindful parenting intervention with another active intervention. Ferraioli and Harris (2013) reported that mindful parenting resulted in a larger reduction in parenting stress than skills-based parent training (*d* = 1.59). Chaplin et al. (2018) reported that mindful parenting outperformed parent education, in two out of the three parenting stress domains measured (*d* = 0.53 and *d* = 0.59). Although not specifically about parenting stress, one study measured parents' heart rate variability and reported an effect of *d* = 0.00 for the comparison between the mindfulness and control groups (Lo et al., 2017b).

### Youth Psychological Outcomes

#### Within-Group Differences

The summary effect sizes for the youth internalizing, externalizing, cognitive, and social domains are presented in Table 4. Post-intervention effect sizes for each domain were small, and all were maintained at 2-month follow-up.

**Table 4:** Within-group effects for four youth outcome domains.

**Outcome domain**	**Point of assessment**	**Sample**		**Effect size**			**Heterogeneity**	
		***K***	***n***	**Hedges' *g***	***p*-value**	**95% CI**	***I*^**2**^**	***p*-value**
Internalizing	Post-intervention	12	438	0.29	<0.001	0.21–0.36	22%	0.229
	Follow-up#	9	397	0.33	<0.001	0.22–0.44	46%	0.065
Externalizing	Post-intervention	14	621	0.26	<0.001	0.18–0.34	37%	0.079
	Follow-up	10	414	0.39	<0.001	0.31–0.47	7%	0.379
Cognitive	Post-intervention	7	231	0.27	0.001	0.11–0.42	52%	0.051
	Follow-up	5	144	0.40	<0.001	0.24–0.55	24%	0.263
Social^∧^	Post-intervention	5	158	0.28	<0.001	0.14–0.43	25%	0.254

K, number of studies included in the effect size calculation; n, total number of participants in the studies included in the relevant domain;

# , all follow up assessments are 2 months post-intervention, except for one study included in the Externalizing domain, which conducted follow-up 4 months post-intervention;

∧ *, follow-up data were not analyzed for the Social outcomes domain, as only three studies reported follow-up social outcome data*.

**Figure 3:**
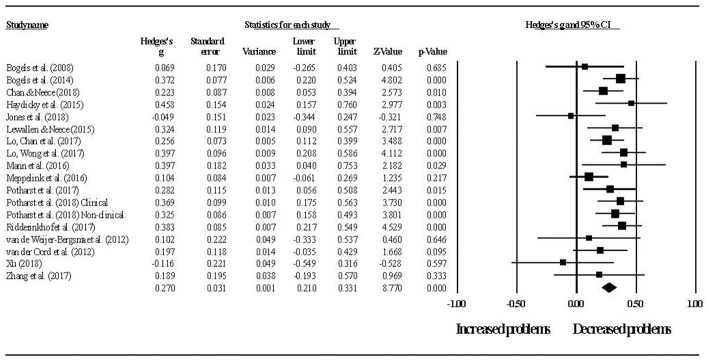
Pre- to post-intervention change in overall youth outcomes.

Figure 3 shows the effect sizes for overall youth outcomes. The summary effect size was *g* = 0.27 (*p* < 0.001, 95% CI [0.21–0.33]), with low to moderate heterogeneity (*Q* = 23.06, *p* = 0.147, *I*^2^ = 26%). At 2-month follow-up, the summary effect was *g* = 0.35 (*p* < 0.001, 95% CI [0.27–0.42]), with low heterogeneity (*Q* = 10.45, *p* = 0.402, *I*^2^ = 4%). There was no difference between pre-post and pre-follow up effects (*Q*_B_ = 2.53, *df* = 1, *p* = 0.112).

Despite the relatively low level of heterogeneity in youth outcome effects, moderator analyses were conducted in respect of youth age (child vs. adolescent) and intervention groups (parent only vs. parallel parent and youth groups). There were insufficient studies to conduct this analysis in respect of parent or youth clinical status. No differences were found in overall youth outcome effect sizes for children (*g* = 0.26, *p* < 0.001, 95% CI [0.20–0.33]) and adolescents (*g* = 0.30, *p* = 0.001, 95% CI [0.13–0.48]) (*Q*_B_ = 0.17, *df* = 1, *p* = 0.682) or for studies using parent only interventions (*g* = 0.26, *p* < 0.001, 95% CI [0.18–0.33]) and studies using parallel parent and youth interventions (*g* = 0.31, *p* < 0.001, 95% CI [0.21–0.41]) (*Q*_B_ = 0.71, *df* = 1, *p* = 0.399).

**Figure 4:**
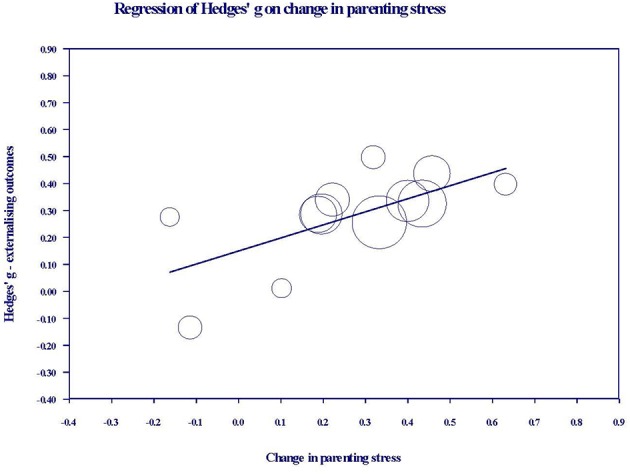
Bubble plot of youth externalizing outcome effects against change in parenting stress. Each bubble represents a study, and the diameter of each bubble is proportional to the study weight.

**Figure 5:**
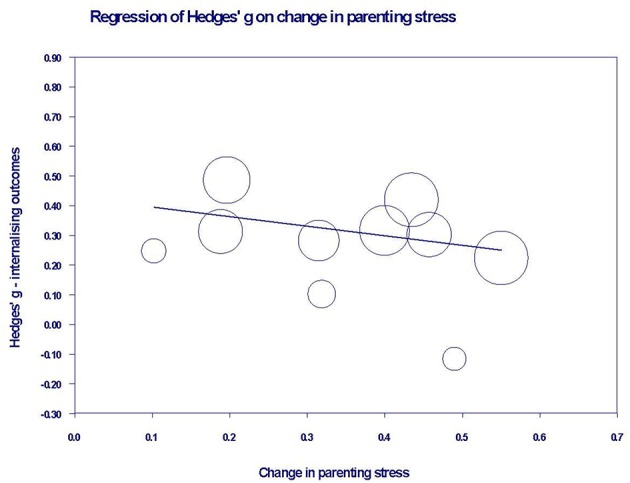
Bubble plot of youth internalizing outcome effects against change in parenting stress. Each bubble represents a study, and the diameter of each bubble is proportional to the study weight.

A meta-regression of total intervention hours on overall youth outcomes was conducted, but no evidence was found of a relationship between these two variables (β = 0.00, *SE* = 0.00, *p* = 0.844). For those studies reporting both parenting stress and youth outcome data, a series of meta-regressions were conducted to examine whether change in parenting stress predicted youth outcome effect sizes. Change in parenting stress predicted change in both youth externalizing (β = 0.48, *SE* = 0.21, *p* = 0.02) and cognitive outcomes (β = 1.13, *SE* = 0.56, *p* = 0.046), but not internalizing outcomes (β = −0.32, *SE* = 0.30, *p* = 0.282). The same analysis was not performed for the social domain as there were too few studies. Figures 4, 5 show the relationships between change in parenting stress and externalizing outcomes, and change in parenting stress and internalizing outcomes, respectively.

Insufficient data was available for a quantitative analysis of youth mindfulness, but the effects reported by five studies for this variable (see Table 3) ranged from *d* = −0.26 to *d* = 0.50. A small number of studies included objective measures of youth outcomes, such as attention tests. In two studies, the effects obtained in the attention tests were broadly in line with those obtained from self-reports. For example, in Bögels et al. (2008), the youth-reported effect for attention problems was *d* = 1.00, then *d* = 0.90 at follow up, while the effect reported based on the D2 Attention Test was *d* = 0.60, rising to *d* = 1.10 at follow up. Similarly, in van de Weijer-Bergsma et al. (2012), the youth-reported effect for attention problems was *d* = 0.50, while the computerized sustained attention task effects ranged between *d* = 0.20 and *d* = 0.40. In Zhang et al. (2017), the effects reported for several aspects of attention were variable. For example, the effects in various subtests of sustained attention ranged from *d* = −0.24 to *d* = 0.76.

Only one study reported mother and father data on youth outcomes separately (van de Weijer-Bergsma et al., 2012), and two studies obtained teacher reports of youth outcomes (Lewallen and Neece, 2015, reported in Table 3 under Neece, 2014; van de Weijer-Bergsma et al., 2012). Teacher-reported effects were similar to parent-reported effects in van de Weijer-Bergsma et al. However, in Lewallen and Neece, teachers reported significant improvements in all seven of the social domains measured, whereas parents reported significant improvements in only three domains.

#### Between-Group Differences

No quantitative comparison of the effectiveness of mindfulness interventions to control groups for youth outcomes was performed, as data required for this analysis was only available for three studies. However, of the studies that reported a between-group effect, the mindfulness group outperformed wait list for externalizing problems in two out of five studies [*d* = 0.29 in Lo et al. (2017b) and *d* = 0.60 in Mann et al. (2016)] and for internalizing problems in one out of three studies [*d* = 0.46 in Lo et al. (2017b)]. There were no studies comparing mindfulness with an active control, for youth psychological outcomes.

### Publication Bias

**Figure 6:**
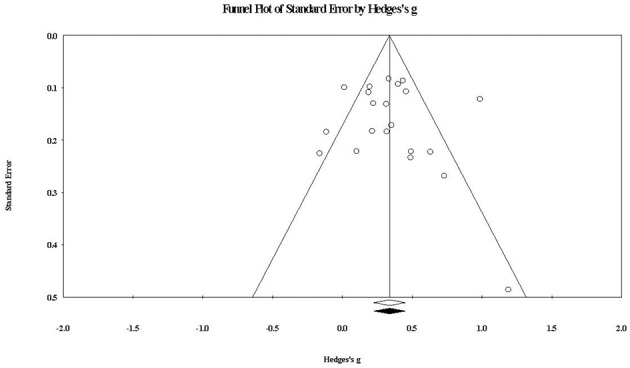
Funnel plot of standard error by within-group parenting stress effect sizes. The white diamond represents the observed summary effect size, while the black diamond represents the imputed summary effect size free of publication bias.

To assess the impact of any publication bias on the observed effects in this review, the trim and fill method (Duval and Tweedie, 2000) was used to give unbiased estimates of effect size. For within-group parenting stress, the imputed summary effect size was *g* = 0.33, which was equal to the observed summary effect size of *g* = 0.33. As shown in Figure 6, the trim and fill analysis indicated that no studies were required to be trimmed in order for the funnel plot to be symmetric, that is for the impact of any publication bias to be removed. In relation to between-group parenting stress, the trim and fill analysis produced an imputed summary effect size of *g* = 0.32 (compared to the observed *g* = 0.35), with one study needing to fall on the left of the summary effect for plot symmetry. The impact of any publication bias in relation to parenting stress effects appears likely to be trivial.

**Figure 7:**
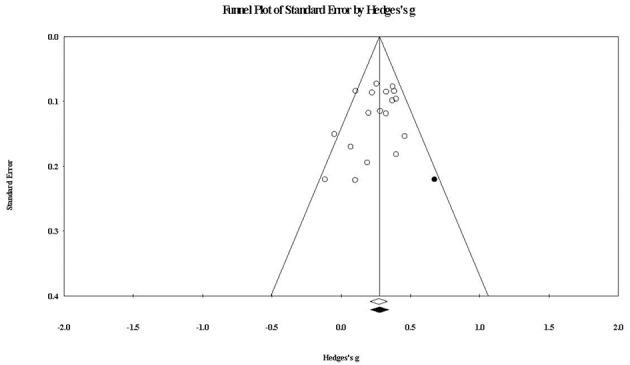
Funnel plot of standard error by within-group overall youth outcomes effect sizes. The black circle represents the effect size of the imputed study that would be required to remove publication bias. The white diamond represents the observed summary effect size, while the black diamond represents the imputed summary effect size free of publication bias.

For within-group overall youth outcomes, the funnel plot at Figure 7 shows that one study would need to fall on the right side of the observed summary effect for plot symmetry. The imputed effect size was *g* = 0.281 (compared to the observed *g* = 0.276), again suggesting a trivial impact of publication bias.

### Assessment of Study Quality

Table 5 contains risk of bias assessments for each reviewed study. Overall, risk of bias was serious. For the non-randomized intervention studies, this was largely driven by the serious risk of confounding bias, which ROBINS-I notes may occur if any prognostic variable also predicts the intervention received by a participant. Due to the lack of randomization, it is considered likely to be an issue for most if not all non-randomized studies (Sterne et al., 2016). For both non-randomized studies and RCTs, the majority of studies were considered at serious risk of detection bias because of the reliance on subjective self- or parent-about-youth outcome reports, which are considered reasonably vulnerable to the influence of knowledge about the intervention. Bias due to potential misclassification was an issue in many studies, as most reports did not state their pre-intervention position as to the minimum number of sessions a participant would need to attend to be considered as having completed the intervention. Bias may be introduced if the minimum number of sessions was changed after the study commenced. Many studies also reported limited information regarding items such as session attendance rates of treatment completers, homework completion and instructor training, making it difficult to properly assess the risk of performance bias.

**Table 5:** Risk of bias assessment for reviewed studies.

**Study**	**Confounding bias^**a**^**	**Selection bias^**b**^**	**Misclassification bias**	**Performance bias**	**Attrition bias**	**Detection bias**	**Reporting bias**
Bazzano et al. (2015)	Serious	Low	Moderate	Unclear	Low	Serious	Moderate
Bögels et al. (2008)	Serious	Low	Moderate	Low	Low	Serious	Moderate
Bögels et al. (2014)	Serious	Low	Moderate	Low	Low	Serious	Moderate
Corthorn (2018)	Serious	Low	Unclear	Unclear	Moderate	Serious	Moderate
Chan and Neece (2018)^#^	–	Low	Unclear	Low	Low	Serious	Moderate
Chaplin et al. (2018)^#^	–	Unclear	Unclear	Unclear	Low	Serious	Moderate
De Bruin et al. (2015)	Serious	Low	Unclear	Low	Low	Serious	Moderate
Eames et al. (2015)	Serious	Low	Low	Unclear	Serious	Serious	Moderate
Ferraioli and Harris (2013)^#^	-	Unclear	Unclear	Low	Moderate	Serious	Moderate
Haydicky et al. (2015)	Serious	Low	Moderate	Low	Moderate	Serious	Moderate
Jones et al. (2018)	Serious	Low	Unclear	Unclear	Moderate	Serious	Moderate
Lewallen and Neece (2015)	Serious	Low	Unclear	Unclear	Moderate	Moderate	Moderate
Lo et al. (2017a)^#^	–	Unclear	Unclear	Low	Low	Serious	Moderate
Lo et al. (2017b)^#^	–	Low	Unclear	Low	Low	Moderate	Low
Maloney and Altmaier (2007)	Serious	Low	Unclear	Unclear	Unclear	Serious	Critical
Mann et al. (2016)^#^	–	Low	Moderate	Low	Moderate	Serious	Low
Meppelink et al. (2016)	Serious	Low	Unclear	Unclear	Moderate	Serious	Moderate
Neece (2014)^#^	–	Low	Unclear	Low	Low	Serious	Moderate
Potharst et al. (2017)	Serious	Low	Unclear	Low	Moderate	Serious	Moderate
Potharst et al. (2018)	Serious	Low	Moderate	Low	Moderate	Serious	Moderate
Racey et al. (2017)	Serious	Low	Moderate	Moderate	Critical	Critical	Moderate
Ridderinkhof et al. (2017)	Serious	Low	Unclear	Moderate	Moderate	Serious	Moderate
Short et al. (2017)	Serious	Low	Unclear	Moderate	Low	Serious	Moderate
van de Weijer-Bergsma et al. (2012)	Serious	Low	Unclear	Low	Moderate	Moderate	Serious
van der Oord et al. (2012)	Serious	Low	Low	Low	Low	Serious	Moderate
Voos (2017)	Serious	Low	Moderate	Unclear	Moderate	Serious	Moderate
Xu (2017)	Serious	Low	Unclear	Unclear	Serious	Serious	Moderate
Zhang et al. (2017)	Serious	Low	Unclear	Moderate	Low	Serious	Moderate

# RCT. For all RCTs in this table, the terms used to describe the level of bias have been changed from “Low,” “High,” and “Unclear” (used in the RoB tool), to “Low,” “Moderate,” “Serious,” “Critical,” and “Unclear,” to reflect the terms and judgment guidelines used in ROBINS-I;

a not relevant for RCTs;

b *For RCTs, the assessment of selection bias asks (1) whether there was random sequence generation and (2) whether there was allocation concealment. In this table, only one risk assessment is reported for RCTs under this bias domain, as the level of risk assessed for these two aspects of selection bias was equal for each of the reviewed RCTs*.

## Discussion

This review examined 25 independent studies of mindfulness interventions delivered to parents. We systematically evaluated the effectiveness of these interventions in reducing parenting stress and improving youth psychological outcomes. The results of the review show that mindfulness interventions for parents are associated with small to moderate immediate and maintained reductions in parenting stress. Reductions in parenting stress are greater for parents who attend mindfulness intervention groups than for those who attend control groups. Results also show that mindfulness interventions for parents are associated with small immediate and maintained improvements for youth across internalizing, externalizing, cognitive, and social domains of psychological functioning. Improvements in youth externalizing and cognitive outcomes are predicted by reductions in parenting stress, but no relationship was found between youth internalizing outcomes and parenting stress. There were insufficient studies to test the relationship between parenting stress and social outcomes.

### Parenting Stress

For parenting stress, the small within-group reduction (*g* = 0.34) obtained immediately after intervention rose to a moderate reduction (*g* = 0.53) 2 months later. This suggests that the positive impact on parenting stress of the mindfulness intervention continued after the intervention ended. Two studies also measured parenting stress 1 year after the intervention, both reporting the maintenance of small to moderate reductions in parenting stress at that point. The five controlled studies reviewed showed that mindfulness interventions have a small to moderate advantage (*g* = 0.44) over active and waitlist controls in reducing parenting stress. These results, together with the finding that pre-test mindful parenting scores are negatively correlated with post-test parenting stress, but not vice versa (Haydicky et al., 2015), provide initial evidence that mindfulness interventions for parents contribute to reduced parenting stress.

To place our findings regarding the parenting stress effect size into context, we sought to compare the current results against those obtained in other meta-analyses. We were unable to find meta-analyses of mindfulness or other interventions that aimed at lowering parenting stress specifically. However, Lundahl et al. (2006a) assessed parental emotional adjustment, which incorporated parenting stress. They reported a moderate within-group improvement in that outcome, in their review of parent programs to reduce child abuse. The post-intervention effect in that study (*d* = 0.53) was larger than in the present study (*g* = 0.34). This may have been because the measure of parental emotional adjustment included a number of negative emotional states, such as anger, in addition to parenting stress. It is therefore possible that the effect size was driven by improvements in emotional states other than parenting stress.

We also sought to compare the advantage we found for mindfulness interventions over control groups to that found for other parent interventions. Again, we were unable to find any published meta-analyses concerning parenting stress as a stand-alone outcome. However, Lundahl et al. (2006b) reviewed the effects of parent training programs on a composite parenting outcome, which included parenting stress. Lundahl et al. (2006b) defined behavioral training programs as those teaching parents to reinforce their children's positive behavior and ignore or punish poor behavior. Non-behavioral programs were defined as those that did not teach these specific skills, and included programs aimed at improving parent-child communication or altering child-related cognitions. Based on this definition, mindfulness interventions are non-behavioral programs, and indeed the advantage over controls in the present study (*g* = 0.44) is similar to that found by Lundahl et al. (2006b) for non-behavioral parent programs (*d* = 0.48). The advantage of behavioral programs over controls was slightly larger (*d* = 0.53).

Interestingly, this review also found that the reduction in parenting stress was greater at follow up than post-intervention. This is in contrast to the pattern reported for behavioral parent training by Lee et al. (2012), who found a reduced effect at follow-up for a composite parenting outcome that included parenting stress. Similarly, the effects of cognitive behavioral therapy for general stress are maintained at follow up, but not increased (Hofmann et al., 2012). The present results suggest, therefore, that mindfulness interventions provide durable outcomes for parents, and compare favorably in this respect to behavioral parent training and cognitive behavioral therapy.

Heterogeneity in relation to parenting stress is moderate to high, indicating variance in the true effect size across studies. Possible reasons for this variability were tested through categorical moderator analyses and meta-regression. The reduction in parenting stress was not moderated by either youth age or clinical status, or the length of the mindfulness course. This suggests that parents acquire generic skills in mindfulness programs lasting from 9 to 27 h, that they are able to apply in various parenting environments, and across their child's development. In contrast, the reduction in parenting stress was greater when the intervention was delivered only to parents, than when it was delivered to parallel parent and youth groups. This result was surprising, since it is reasonable to expect that training both parents and their children in mindfulness would contribute to better outcomes, given the bi-directionality of parent and child factors (Branje et al., 2010; Neece, 2014). To investigate this result further, the characteristics of the two subgroups were checked. Of the six studies in the parallel interventions subgroup, five involved youth diagnosed with ADHD. However, amongst the 15 studies in the parent-only intervention subgroup, only three involved parents whose children had been diagnosed with ADHD. Further, these three studies reported only 47, 31, and 7% of the parents' children as having ADHD. While no conclusion can be drawn, it is possible that the smaller reduction in parenting stress amongst parents in the parallel intervention subgroup is related to their child's diagnosis of ADHD, rather than the fact that both parents and their children received the intervention.

### Youth Outcomes

The results of our review show that mindfulness interventions for parents are associated with improved youth outcomes. The summary effects indicate small, within-group improvements in internalizing (*g* = 0.29), externalizing (*g* = 0.26), cognitive (*g* = 0.27), and social (*g* = 0.28) domains. These improvements are maintained after 2 months for the internalizing (*g* = 0.33), externalizing (*g* = 0.39), and cognitive (*g* = 0.40) domains. There were insufficient studies to conduct a follow-up analysis for the social domain. There were also insufficient controlled studies to conduct a quantitative comparison of intervention groups with controls, for any of the youth outcomes. The results reported by the few studies that included a control group are mixed, with mindfulness groups outperforming waitlist controls in some studies but not others, for both internalizing and externalizing outcomes.

This is the first published meta-analysis regarding the effectiveness of mindfulness interventions for parents in improving youth outcomes. There are, therefore, no equivalent studies to compare the effects found in the present review against. A review of mindfulness interventions delivered to children and adolescents in schools found within-group effects for emotional problems and cognitive performance of *g* = 0.31 and *g* = 0.68, respectively (Zenner et al., 2014). It is possible that the effects reported in that study were larger than those in the present review because the interventions were delivered directly to the children and adolescents, rather than to parents. Looking at other parent-focused interventions, a meta-meta-analysis of studies for treating youth with externalizing disorders obtained effects for youth outcomes (externalizing and internalizing problems combined) of *d* = 0.46 post-intervention and *d* = 0.49 at follow-up (Mingebach et al., 2018). The larger improvements found in that review may reflect the fact that the majority of reviewed studies involved behavioral parent training interventions. Mindfulness interventions for parents appear, therefore, to be associated with smaller improvements in youth outcomes than either behavioral parent training or mindfulness interventions for youth.

Heterogeneity in connection with youth outcomes is low to moderate. Mindfulness interventions for parents are associated with equally beneficial outcomes for children and adolescents, whether they attend mindfulness training in parallel with their parents or not, and regardless of the length of the mindfulness course. These results together suggest that even shorter mindfulness programs can result in changes to parental functioning that are positive for youth of any age. Meta-regressions were conducted to check whether change in parenting stress predicted youth outcomes. Greater reductions in parenting stress did predict greater improvements in youth externalizing and cognitive outcomes. This finding is consistent with previous studies showing that parenting stress is related to harsh, over-reactive parenting (Venta et al., 2016), and that harsh parenting predicts later youth behavior problems and poorer attentional regulation (Eisenberg et al., 1999; Rominov et al., 2016). Therefore, reductions in parenting stress may improve externalizing and cognitive outcomes.

Unlike externalizing and cognitive outcomes, reductions in parenting stress did not predict improvements in youth internalizing outcomes. There are a number of possible explanations for this. While youth externalizing problems can be aversive to parents and contribute to higher parenting stress (Eisenberg et al., 1999; Neece et al., 2012), youth internalizing problems tend to be subtle and non-aversive (Eisenberg et al., 1999). Accordingly, it is possible that parents of youth with internalizing problems have a lower baseline level of parenting stress than do parents of youth with externalizing problems. In this case, we would expect a mindfulness intervention for parents of youth with internalizing problems to have less of an impact on parenting stress. Any relationship between change in parenting stress and change in internalizing problems may therefore be too small to detect. Mindfulness interventions for parents could also affect youth internalizing outcomes through a pathway other than parenting stress. For example, greater parental warmth and acceptance toward children are associated with lower youth internalizing problems (Yap and Jorm, 2015). As mindful parenting involves compassion, emotional warmth, and non-judgmental acceptance toward a child (Duncan et al., 2009, 2015), mindfulness interventions may improve internalizing outcomes by promoting these attitudes in parents. Internalizing problems are also associated with difficulties with emotion regulation (Suveg and Zeman, 2004). For example, greater use by parents of adaptive emotion regulation strategies, such as cognitive reappraisal, are associated with lower youth anxiety (Wald et al., 2018). Since mindful parenting is also associated with greater parental self-regulation (Duncan et al., 2009; Ridderinkhof et al., 2017), mindfulness interventions could reduce youth internalizing problems by facilitating healthier forms of emotional regulation in parents.

### Methodological Limitations

There are several limitations affecting the strength of the evidence provided by both this review and the individual studies reviewed. At the review level, the number of studies available for inclusion is still small. For this reason, we treated studies of mindful parenting interventions and studies of other mindfulness-based interventions delivered to parents as a single group. However, it is not currently known whether these two types of mindfulness intervention have different outcomes for parents or youth, or whether they exert their effects through different pathways. The number of available studies also had implications for testing potential moderators, such as parent clinical status. It may also have affected our ability to detect significant moderators and covariates. For example, although we found no relationship between the length of the mindfulness course and either parenting stress or youth outcomes, some other meta-analyses have found dose-response relationships for a range of outcomes (Khoury et al., 2013; Zenner et al., 2014; cf. Vollestad et al., 2012). In general, due to the relatively small number of studies in this review, some caution should be applied to the interpretation of the moderator and meta-regression analyses. As more research is published on mindfulness interventions for parents, future reviews with greater power will provide more accurate information regarding significant moderators or covariates.

At the individual study level, small sample sizes are likely to have contributed to a lack of statistical power to detect significant effects in a number of studies. A scan of Tables 2, 3 reveals several moderate to large effects, both post-intervention and at follow-up, that are reported as non-significant. The availability of small samples may have been a reason for the single group design used in most of the reviewed studies. Due to the lack of randomization to intervention or control groups, we cannot conclude that the reported effects are caused by the mindfulness intervention. This is particularly the case for the various outcomes (anxiety, depression, well-being, rumination, and executive functioning) that significantly improved at follow up, but not immediately post-intervention. This longer term effect is consistent with the self-sustaining change proposed to be the result of mindfulness practice (Dumas, 2005). However, childhood is an ongoing period of development in which changes may occur in various domains of functioning over time, for many reasons. When more time has passed, it is more likely that extraneous variables may have contributed to changes in outcomes, making the causal link between the intervention and the effect more tenuous.

All studies were judged to have at least a serious risk of bias. Whilst this was partly due to the lack of randomization noted above, the subjective reporting of most outcomes in each study was also an issue. In the context of mindfulness interventions, which parents must invest a significant amount of time and effort to attend, relying on parent reports may increase the risk of detection bias. Although it is difficult to address this issue in studies in which many outcomes must be subjectively reported, obtaining reports from different sources, such as mothers, fathers, youth and teachers, and obtaining objective measures if possible, may give a more complete picture. For example, Lewallen and Neece (2015) found that teachers reported significant improvements in more social domains than parents did. This suggests that youth outcomes may differ across contexts. Similarly, the differences between mothers and fathers in post-intervention parenting stress (van de Weijer-Bergsma et al., 2012) might indicate a systematic difference in how mothers and fathers respond to a mindfulness intervention. Finally, assessment of treatment adherence and integrity was problematic in many studies, as limited information was reported regarding session attendance rates, homework completion or instructor training. Lack of detailed implementation-related data appears to be a common issue in connection with mindfulness interventions (Vollestad et al., 2012; Zou et al., 2018).

### Future Directions

The results of this review show that further research on mindfulness interventions for parents is desirable. Future studies are needed to address the methodological limitations identified above. For example, there is evidence that variables such as therapist experience with mindfulness (Khoury et al., 2013), amount of home practice (Parsons et al., 2017) and total time of mindfulness training (Zenner et al., 2014) can moderate outcomes. Inclusion of more information on these variables would allow reviewers to investigate more potential moderators. In addition, randomizing participants to control and intervention groups would allow firmer conclusions to be drawn about whether mindfulness in parenting played a causal role in relevant outcomes.

Use of randomized controlled studies would also allow comparisons to be made between mindfulness interventions and other active interventions such as behavioral parent training. For youth with externalizing problems, behavioral parent training is an effective and widely used intervention (Dretzke et al., 2009). However, some parents, such as those with their own psychopathology, benefit less from behavioral parent training than others (Maliken and Katz, 2013). This may be because these parents find it difficult to apply new parenting skills in stressful situations with their child and revert to old patterns of responding in those situations (Siegel and Hartzell, 2004). Given its focus upon reducing parenting stress, mindfulness-based interventions might be of greater benefit to these families than behavioral parent training.

The majority of studies involved parents with children under 12 years, or parents managing youth externalizing problems. Very few studies included parents of youth with internalizing problems. It is therefore recommended that additional research be done in community samples or in clinical samples of families experiencing youth internalizing problems. As no relationship was found between parenting stress and youth internalizing outcomes, research with these samples could investigate whether mindfulness in parenting is associated with potential mediators other than parenting stress. These could include parental factors known to be associated with youth internalizing problems. Finally, relatively few studies examined outcomes for families with adolescents and only one of these (Corthorn, 2018) included parents of adolescents without a clinical diagnosis. Adolescence is associated with increased negative affect (Kim et al., 2001) and conflict (Laursen et al., 1998), and may be a time of potentially stressful change in the parent-child relationship (Duncan et al., 2009). Importantly, it is also a time when many psychological disorders are first diagnosed (Copeland et al., 2009). Research could usefully address the question of whether mindfulness interventions for parents of adolescents are effective as a preventive intervention for adolescent psychological problems.

## Conclusion

The results of the present review show that mindfulness interventions for parents are associated with reduced parenting stress for parents of both children and adolescents. They are also associated with improved youth psychological functioning across internalizing, externalizing, cognitive, and social domains. Reduced parenting stress predicts improvement in youth externalizing and cognitive outcomes, but not youth internalizing outcomes. Methodological weaknesses in the available literature prevent firm conclusions from being drawn regarding the causal role of mindfulness training for parents in relation to each of these outcomes. Further research is recommended to address limitations in the current literature and questions raised by this review.

## Author Contributions

VB designed and conducted the review and meta-analysis and wrote the manuscript. MS and MA reviewed the design and collaborated on editing the manuscript.

### Conflict of Interest Statement

The authors declare that the research was conducted in the absence of any commercial or financial relationships that could be construed as a potential conflict of interest.
